# Associations between biomass fuel use and child health: a community-based study in Bhaktapur, Nepal

**DOI:** 10.1093/eurpub/ckac130.036

**Published:** 2022-10-25

**Authors:** C Schwinger, I Kvestad, RK Chandyo, M Hysing, M Ulak, M Shrestha, S Ranjitkar, L Shrestha, TA Strand

**Affiliations:** 1 Centre for International Health, University of Bergen, Bergen, Norway; 2 NORCE, Bergen, Norway; 3 Department of Research, Innlandet Hospital Trust, Lillehammer, Norway; 4 Department of Community Medicine, Kathmandu Medical College, Kathmandu, Nepal; 5 Department of Psychosocial Science, University of Bergen, Bergen, Norway; 6 Department of Child Health, Tribhuvan University, Kathmandu, Nepal; 7 Centre for Disease Burden, Norwegian Institute of Public Health, Bergen, Norway

## Abstract

**Background:**

Biomass fuel use for cooking is widespread in low- and middle-income countries. Previous studies have mainly focused on adverse health outcomes in adults or specific diseases. In a cohort among young children living in Bhaktapur, Nepal, we aimed to describe the association between the use of biomass cooking fuels in families with child health using measures of linear growth, cognition and chronic illness.

**Methods:**

Caregivers of 600 marginally stunted children aged 6-11 months were interviewed about their primary source of cooking fuel at enrolment into a randomized controlled trial. Children's body length (n = 572) was measured at age 18-23 months. At the same time, blood samples (n = 497) were taken, and we measured leukocyte telomere length (LTL) as a marker of chronic disease risk. We chose LTL expressed as z-scores as a measure of chronic disease. Cognitive abilities were measured by the Wechsler Preschool and Primary Scale of Intelligence, 4th edition (WPPSI-IV) and NEPSY-II subtests when the children were 4 years old (n = 531). Associations were estimated in multiple regression models.

**Results:**

About 18% of all families used biomass as primary cooking fuel. Children from families using biomass fuel were on average slightly shorter (mean difference 0.14 Z-scores, 95% CI: 0.28, 0.00), had lower IQ scores (mean difference 2.2 (95% CI: 0.5, 3.9), and shorter LTL (mean difference: 0.09 (95% CI: 0.05 to 0.13) compared to those not using biomass fuel. The observed associations were unaltered after adjusting for relevant confounders.

**Conclusions:**

In children from households in poor, urban neighborhoods in Nepal, biomass fuel use for cooking was associated with health indicators for child growth and cognition as well as longevity and chronic illnesses reflected in shortening of telomeres. As this was an observational study, residual confounding cannot be excluded. Our findings support the ongoing effort to reduce exposure to biomass fuel in low-resource settings.

**Key messages:**

- Biomass fuel use was associated with diverse child health indicators in young Nepalese children.
- A better understanding of mechanisms leading to adverse health outcomes early in life is needed.

